# Social representation of palliative care in the Spanish printed media: A qualitative analysis

**DOI:** 10.1371/journal.pone.0211106

**Published:** 2019-01-25

**Authors:** José Miguel Carrasco, Beatriz Gómez-Baceiredo, Alejandro Navas, Marian Krawczyk, Miriam García, Carlos Centeno

**Affiliations:** 1 ATLANTES Research Program, Institute for Culture and Society, University of Navarra, Pamplona, Spain; 2 Journalism Projects Department, School of Communication, University of Navarra, Pamplona, Spain; 3 Public Communication Department, School of Communication, University of Navarra, Pamplona, Spain; 4 Centre for Health Evaluation and Outcome Sciences, Vancouver, Canada; 5 Trinity Western University, Langley, Canada; 6 Errea Comunicación, Pamplona, Spain; 7 Clínica Universidad de Navarra, University of Navarra, Pamplona, Spain; 8 Navarra Institute for Health Research (IdiSNA), Pamplona, Spain; Lancaster University, UNITED KINGDOM

## Abstract

**Background:**

Lack of social awareness is a major barrier to the development of palliative care. Mass media influences public opinion, and frequently deal with palliative care contributing to its image and public understanding.

**Aim:**

To analyse how palliative care is portrayed in Spanish newspapers, as well as the contribution made by the press to its social representation.

**Design:**

Based on criteria of scope and editorial plurality, four print newspapers were selected. Using the newspaper archive MyNews (www.mynews.es), articles published between 2009 and 2014 containing the words “palliative care” or “palliative medicine” were identified. Sociological discourse analysis was performed on the identified texts on two levels: a) contextual analysis, focusing on the message as a statement; b) interpretative analysis, considering the discourse as a social product.

**Results:**

We examined 262 articles. Politician and healthcare professionals were the main representatives transmitting messages on palliative care. The discourses identified were characterised by: strong ideological and moral content focusing on social debate, strong ties linking palliative care and death and, to a lesser degree, as a healthcare service. The messages transmitted by representatives with direct experience in palliative care (professionals, patients and families) contributed the most to building a positive image of this healthcare practice. Overall, media reflect different interests in framing public understanding about palliative care.

**Conclusion:**

The knowledge generated about how palliative care is reflected in the printed media may help to understand better one of the main barriers to its development not only in Spain, but also in other contexts.

## Introduction

Due primarily to the reduction of premature mortality and the control of communicable disease, the majority of countries worldwide have experienced a significant increase in life expectancy and the ageing of their populations. As a result, the risk of developing cancer and neurodegenerative diseases has increased and the prevalence of chronic disorders and diseases with lengthy trajectories is expected to rise in the coming years [1–3]. Managing this demographic and epidemiological situation represents a major public health challenge. Palliative care (PC) has repeatedly been proposed as a key element for successfully managing these concerns, with institutions such as the World Health Organization (WHO) and the World Palliative Care Alliance repeatedly highlighting the importance of developing and integrating PC in healthcare systems [2,4].

Palliative care has been defined by the WHO as “*an approach that improves the quality of life of patients and their families facing the problem associated with life-threatening illness*, *through the prevention and relief of suffering by means of early identification and impeccable assessment and treatment of pain and other problems*, *physical*, *psychosocial and spiritual”* [5]. In the 1980s and 1990s PC expanded globally, with approximately half of the countries worldwide having established hospice or PC services. Evidence indicates that much work remains to be done; in many European countries services are still insufficient to meet the needs of the population and falls well below population-based guidelines [6]. In Spain, PC was first implemented in the late 1980s and has been slowly integrated within the national public health care system, as well as in the private context, and currently there are close to 300 PC resources in the country [7]. Nonetheless, in 2012 PC coverage remains at less than half of the recommended amount, and the level of service development remains insufficient to cover the needs of the Spanish population [6].

Challenges to the growth of PC include constraints on financial and material resources, limited availability and knowledge about opioids, few PC training and education programs, and the lack of public awareness [8,9]. In Spain, challenges include the traditional health services approach focussing on curative and resuscitative objectives, in combination with cultural taboos about discussing end-of-life and the lack of knowledge and awareness about PC. Therefore the development of PC in Spain may be significantly affected by social perceptions [10,11].

Mass media in general shapes the ideas that circulate in ‘public space’, defined as the social environment where public opinion is negotiated [12]. The mass media generates and reflects existing social discourses, defined as the practices through which a message is transmitted using means of expression such as symbols, narratives, metaphors, and other signs that give meaning to reality [13]. In turn, mass mediated messages have the potential of creating public opinion through the discourses they shape and negotiate [14–16]. These discourses have power to fuel the social imaginary and mediate individuals’ beliefs and lived experiences, including significant influence in shaping public perceptions and societal attitudes concerning health, illness, and consequently, health care services [17]. While previous research has explored print media representations of health concerns [18,19], to our knowledge there is little focused on PC and its influence in terms of generating the social image of these services [20].

An analysis of mass media news items using qualitative research techniques may help to understand and interpret the meaning given to PC [17,21]. The aim of this study is to examine how PC is portrayed in the Spanish mainstream print media, as well as the contribution made by the press to its social representation.

## Materials and methods

We undertook a qualitative study using sociological discourse analysis to examine mainstream media representations of PC. Four Spanish general printed newspapers were selected based on their readership, scope and editorial plurality aiming to have a global vision on how PC is portrayed in the Spanish context: *ABC*, *El País*, *El Mundo* and *La Vanguardia*. The first three are published in Madrid and the last is published in Barcelona. Ideologically, *ABC* is conservative, *El Mundo* and *La Vanguardia* are in the centre and *El País* is progressive. Using the Spanish newspaper archive MyNews (www.mynews.es), articles were searched for containing the words “palliative care” or “palliative medicine” (in Spanish “cuidados paliativos” or “medicina paliativa”) that were published in any printed edition between January 2009 and February 2014. The complete text of the news item containing the searched words was obtained and entered into a database.

Sociological discourse analysis (SDA) has been shown as a useful methodology to analyse cultural and social constructions of reality. SDA focuses on the discourses or discursive fields of structured contexts where a set of activities of collective actors is conceived to produce and reproduce reality [13,22]. We undertook a SDA on two levels in the identified texts: *contextual analysis*, focusing on the message as a statement and considering the discourse as a unique fact or occurrence; and *interpretive* analysi*s* by considering the discourse as a social product. These levels of analysis should not be understood as a linear process, but as multi-directional and carried out simultaneously [13]. Discursive positions (who produced the discourse, under what circumstances, with what objective) were also identified.

With the aim of strengthening interpretive results, the analysis was triangulated by three researchers with different backgrounds but with experience in qualitative research: a sociologist with expertise in public health (JMC), a sociologist with expertise in communication (AN) and a communication science professional with expertise in narration (BG). Throughout the study process, the researchers considered reflexivity and self-monitoring in their role as researchers. Descriptive coding categories were mutually generated and agreed on by all three researchers. NVivo software was used for storage of data and for analytic purposes.

This study did not involve human participation and all documents analysed are part of the public domain; it is therefore exempt from ethics approval.

## Results

A total of 295 news items were identified containing the terms searched, and after eliminating duplicate news items and those in which the terms were not correctly identified, 262 articles were included in the study: 135 in *ABC*, 65 in *El País*, 24 in *El Mundo* and 38 in *La Vanguardia* (S1 File). A quantitative description of the news items identified can be found in an earlier article showing that most of the news items were published in the newspapers’ ‘Culture and Society’ (38%) and ‘National’ sections (30%), followed by ‘Editorial’ and ‘Reader’s letters’ (19%) and other sections (12%), with only two per cent appearing in the ‘Health’ section [23]. Due to this variability, the diversity of the language, how it was structured and the graphics resources used was conditioned by the type and scope of each article.

### Context

During the time period analysed, several pieces of regional legislation about end-of-life care were proposed, debated, and passed [24–26]. These contained references to PC and generated both legislative and public debate. Additionally, in 2008–2011 the Spanish government was controlled by the progressive Spanish Socialist Workers’ Party (PSOE), who presented a bill to regulate an individual’s rights at end of life, although the legislation did not pass. This governmental debate was discussed in print newspapers:

*“Aragon passes its own law weeks before national government presented the law*. *The conservative Popular Party (PP) refers to it as a trap to pave the way for assisted suicide (…). ‘Only national legislation can regulate the most pressing matters of palliative care’”*.

Yolanda Aznar; “Socialist regions revive debate on death with dignity and euthanasia”; *ABC*; 25/03/11.

During this time there were also several high-profile cases of people requesting medical assistance to end their lives, as well as court cases initiated by family members. In these instances, appeals for the use of PC were identified both among those in favour and those against assistance requests. Additionally, the controversial *Leganés Case* was still in the news during the study period, which involved doctors being investigated for providing high-dose sedations resulting in the death of terminal patients.

*“The Socialist government passed a law on palliative care of terminal patients, the so-called ‘death with dignity law’, to prevent other cases like the one at (…) Severo Ochoa Hospital in Leganés (Madrid) (…), involving alleged irregular sedation of 400 patients, as acknowledged by the Secretary of the Socialist organization (Spanish Socialist Workers’ Party*, *progressive)”*.

G. Sanz; “PSOE admits that the Montes Case inspired its ‘death with dignity’ law”; *ABC*; 23/11/10.

Debates and new legislation in other countries, such as the legalization of euthanasia in Belgium, were also reflected.

*“(…) Organizations such as Care Not Killing and the Catholic Church are against this position. ‘We expressed our reservations about the depenalization of euthanasia back in 2002, mainly because excellent palliative care is currently available’*, *argued the Archbishop…”*.

Javier Gallego; “Belgium legalizes euthanasia for children”; *El Mundo*; 14/02/14.

Between 2009 and 2014, several civil institutions launched right-to-life campaigns and other public awareness campaigns. News items appeared in the press about them, with some referencing PC.

*“The third right-to-life campaign by Spanish bishops (…) presented a ‘standard’ campaign with ‘a different feel’ focused on the fight against euthanasia shortly after the government (…) announced plans to regulate palliative care for terminally ill patients”*.

Alicia Rodríguez de Paz; “Bishops now focus their attacks on euthanasia”; *La Vanguardia*; 17/03/11.

In these examples PC formed part of the *immediate present context*–a reflection of what was happening within a specific moment in time. References to PC were also grounded within a more *extensive present co*ntext–as a topic of permanent human interest not necessarily related to the immediacy of news items.

### Actors and discursive positions

We identified five social actor groups (stakeholders) represented in the newspaper articles: 1) politicians, 2) health care professionals, 3) civil society organizations, 4) individuals, and 5) journals and editorial teams.

The most active voice regarding PC was that of politicians. When the news articles referenced these stakeholders, what was visible was the instrumental use of PC, where politicians had referenced to frame larger political and managerial strategies that defended their ideological perspective, proposals, and actions of their respective parties. Further, references from this group of stakeholders were dependant on the debate that had captured media attention in the immediate present.

*“Yesterday, the First Vice President (…) listed the 26 laws the government considers a priority and that his government will aim to pass (…) They include the future Palliative Care and Death with Dignity Act (…) made a point to say that it will not be ‘a law about euthanasia’, but will regulate the death with dignity of ‘terminally ill patients’ who are ‘expected to go through a terrible ordeal before dying’”*.

EFE; “Rubalcaba says Death with Dignity Law will be passed in four months”; *El Mundo*; 20/11/10.

To a lesser degree, articles about PC referenced healthcare professionals’ perspectives. They were brought in as key sources of objective information on technical matters in the immediate present, mainly in controversial cases and to discuss laws on end-of-life-care. Their presence was also relevant in news items covering the more extensive present, with feature articles and interviews in which educational messages were transmitted. In addition to professionals in the field of PC, professionals from other healthcare specialities also appeared in the news items.

*“‘We try to control the physical symptoms, such as pain, dyspnea and nausea, so the person lives the situation with as much dignity as possible. We also address the patient’s psychological, emotional and spiritual suffering in collaboration with the family’, said (…)”*.

M.J.P.; “Three thousand patients receive palliative care each year”; *ABC*, Seville edition; 02/11/13.

Civil society stakeholder groups appeared in the discourses and messages containing references to PC through representatives of right-to-life associations and organizations, associations that defend euthanasia, patients’ associations, and the Catholic Church. Similar to politicians, references in the media from these stakeholders tended to focus on the groups’ own ideological, moral and group interests.

*“Yesterday, the Cardinal (…) defended palliative care “for pain”. About the announcement to pass a law on death with dignity, he said ‘the best way to prepare for death is to live with absolute dignity’”*.

Staff writers; “Cardinal Amigo defends palliative care”; *La Vanguardia*; 23/11/10.

References to civil society groups were also found in the newspapers’ Letters to the Editor sections, where readers discussing PC referenced them either in relation to political and social debates, or included them when acknowledging and showing appreciation for the work of PC professionals.

The media included very little individual patient or family member perspectives about PC. When referenced, their contributions mainly consisted of true stories in which they benefited from it as a healthcare service, and messages were structured in the more extensive present than the immediate present.

*“This palliative care centre has around forty patients. (…) She just came back from rehabilitation. She’s happy. She’s been in here for a year and says she feels ‘healthier’ every day. ‘I move better and breathe better …’, she said”*.

María R. Sahuquillo; “Palliatives for dull pain”; *El País*; 02/03/10.

Finally, the journalists and editorial teams also discussed PC in opinion columns and editorials. Their discourses supported the ideological positions and approaches of each newspaper, mainly in relation to the immediate present.

### Discourses

In the political terrain, discourses defending and promoting PC were observed from all ideological positions, although based on widely different arguments. Conservatives primarily focused on ensuring that PC was framed as against euthanasia and assisted dying, and primarily used moral arguments to frame the distinction. While discourses associated with progressive political positions framed PC as separate from euthanasia practices, they also attempted to locate PC within broader contexts of individual rights, and primarily used technical and legal arguments for its promotion and development.

*“Belén Pajares (PP; Popular Party, conservative) said she was sorry that the final goal of the debate was not palliative care but active euthanasia, for which there is ‘no social demand’, she said”*.

I.A./J.G.; “Catalan Parliament agrees to open debate on euthanasia”; *ABC*; 19/06/09.

“The Minister (from PSOE, Spanish Socialist Workers’ Party, progressive) insisted that a great deal of progress had been made on the final text and mentioned the ‘ethical obligation’ of improving the quality of and access to palliative care, ‘a right of every citizen, not a privilege’. But he did not go so far as to pronounce the word ‘euthanasia’, as he was prompted to do by the senator from Mixed Group”.

N. Ramírez de Castro; “Pajín announces controversial death with dignity law by May”; *ABC*; 08/04/10.

A moral discourse was identified in the social debate on euthanasia in which PC was presented as an alternative to euthanasia or at least an option to be considered. In this discourse, the Catholic Church was frequently referenced, as well as statements of their representatives and those of other anti-euthanasia associations and institutions.

*“After an initial positive statement from Cardinal Antonio María Rouco Varela on the law on palliative care, the Catholic hierarchy issued a highly critical press release in which it amended that position. It mentions a possible cover-up of euthanasia and, more significantly, it announces that Catholics should defend the Church’s position in this matter “using all legitimate means”*.

Enric Juliana; “Zapatero and Rajoy face off in dramatic moment for Europe”; *La Vanguardia*; 28/06/11.

PC was frequently associated with the very end of life and was generally tied to inevitable, fearful and painful aspects of dying. When stakeholders with PC experience were referenced they did not challenge this link, however they also highlighted how PC can support the dignity of patients who are sick enough to die, regardless of diagnosis or age.

*“Not only terminally ill cancer patients need help dying with dignity. People in the last stage in life who are suffering from a non-cancerous disease, regardless of their age, also need help. That’s why the lawmakers met yesterday (…) and decided to extend this care to all patients who need it…”*.

Celeste López; “Children also receive palliative care “; *La Vanguardia*; 10/12/10.

An instructional discourse was also evidenced in referencing health professionals with experience in PC who promoted PC by providing information within an educational approach. In contrast to politicians’ messages embedded within controversial cases, the messages of health care professionals highlighted the need to apply a holistic approach when caring for patients in advanced stages of illness. They also called for greater development and promotion of PC. This discourse was largely conditioned by the fact that these professionals were called on as sources of information and opinion in the immediate present, mainly as representatives of their professional association.

*“The government’s announcement brought immediate reactions. The Spanish Society of Palliative Care (SECPAL) applauded the government’s decision because this law will allow for ‘better end-of-life care, as well as a set of rights and freedoms for people in this situation’. The president trusts that healthcare is handled in these cases ‘not just in the physical context’, because ‘it won’t be successful if it’s not combined with treatment of symptoms using social and psychological methods and addressing the meaning of life’”*.

Celeste López, Ana MacPherson; “Death with dignity by law”; *La Vanguardia*; 20/11/10.

However, healthcare professionals did not always steer entirely clear of the debates linked to PC.

*“About 1% of all terminally ill patients, which represents about 2,000 people, request to be euthanized by their doctors each year, and that includes those who are well cared for in palliative care units. This statistic was made public yesterday by the president of the Spanish College of Physicians (OMC) (…) and the president of the OMC’s Central Committee on Ethics (…)*. *Because the percentage is low, the representatives of the College of Physicians believe that the absolute priority at this time is to extend the use of palliative care.” (…)*

Emilio de Benito; “About 2,000 terminal patients request euthanasia per year”: *El País*; 07/01/09.

Politicians and healthcare professionals were united in their discursive position when they used a rights-based discourse focused on equality and right to access PC.

*“About 200,000 people suffer from a terminal disease each year in Spain. But not all of them receive the same treatment. As mentioned yesterday by Javier Rocafort, the President of the Spanish Society of Palliative Care (SECPAL), there are only 400 specialized palliative care units and they are very poorly distributed. (…) he pointed out three other areas of inequality: the difference between cancer patients and other patients (palliative care was traditionally developed to care for cancer patients); the problem of people who live in rural areas, where providing home care is difficult; and age (fewer services are available for children)”*.

E. de B.; “There are only 400 palliative care units for 200,000 terminally ill patients”; *El País*; 29/05/09.

This rights-based discourse was supported and advanced by press releases from the La Caixa Banking Foundation, which sponsors a major programme for care of people with advanced diseases. Articles on the Foundation’s actions appeared in all four newspapers (*La Vanguardia* also included institutional advertising for the institution).

In some cases, PC primarily appeared as descriptive context within a relatively neutral discourse with no reference to moral frameworks, contemporary social debates, medical contexts, or legislative undertakings.

*“(…) staying at the Military Hospital*, *where he was only given palliative care, was considered unnecessary and a place outside Caracas was chosen (…)”*

Emili J. Blasco; “Doctors send Chávez home due to development of a new tumour”; *ABC*; 01/03/13.

The term “palliative care” was used as a metaphor mainly in opinion pieces and editorials. In these instances it was used to describe a lack of eagerness to address problems or where problems had dramatic or unresolved consequences.

*“(…) the political deadlock is blocking some dramatic decision-making (…) the economy has not reacted to palliative care and society is about to consume its backup reserves”*.

Ignacio Camacho; “State of depression”; *ABC*; 28/06/11.

## Discussion

This study shows the results of a sociological discourse analysis applied to references of PC in Spanish print newspapers between 2009 and 2014. Our analysis found that print media references were mainly related to current socio-political debates in the immediate present regarding proposed end-of-life legislation and controversial legal cases. Politicians and health professionals were the main actors providing messages on PC, with politicians lending an ideological political or moral slant to their discourse, and health professionals framing theirs through educational information, which is consistent with the results of a quantitative study of news coverage related to PC in written media (printed and digital) in Spain [23]. Although the political and moral discourses at times referenced the benefits of PC, these messages were entangled in ideological debates and controversies. This entanglement tended to intertwine PC with political and religious meanings unrelated to its definition [5]. Frequent mention of PC in connection with euthanasia and strong links to very end of life may eclipse its benefits in the minds of readers. In turn, this image of PC may generate barriers to its development as those influenced by these representations may avoid making contact with, or request, this form of medical care [27].

The messages transmitted by actors directly involved in PC (professionals, patients and families) contributed the most information about PC, and used a relatively neutral educational discourse to explain and promote PC. Healthcare professionals were referenced for their expertise. Patients and relatives were referenced based on their lived experience through first-person accounts that reflected on the benefits of PC. These “personal” discourses have significant potential to produce positive social representations, as readers can see themselves reflected in the story presented. ‘Reality’ is built socially and the construction of reality begins with life itself [15].

Overall, the presence of PC in the print media in Spain depended on an information agenda of the immediate present, which generally pursued new and notorious items. This meant that PC was often thrust into the heart of debates in which itself was not the topic of discussion, but rather used support a particular ideological position or argument. Each newspaper’s editorial line (progressive or conservative) conditioned the number of news items and opinion pieces printed on the topic and how it was addressed. For example *El País* published articles primarily related with the Leganés Case, while *ABC* favoured statements by Catholic authoritie*s*. This further polarized understanding of PC, where representations were used to justify different positions: rational messages based on science and law (*El País* and *La Vanguardia*), emotional messages steeped in religion and morality (*ABC*), and in more aseptic descriptions of PC services and healthcare practice (*La Vanguardia*). Overall, our results indicate the media does not present a unified understanding of PC, and reflect different interests in framing public understanding about them.

### Strengths and weaknesses of the study

Although digital media is increasingly used to access a wide range of information and perspectives, in the context of Spain the print media is still an important channel through which citizens inform themselves. This means that Spanish newspapers still have significant capacity to influence public opinion [14]. We chose newspapers with divergent ideological positions with the aim of obtaining a broad-based vision of the presence of PC in the Spanish press; furthermore, the chosen newspapers are the most relevant at national level with close to 4 million readers per day in 2014 (El País: 1.612.000; El Mundo: 1.006.000; La Vanguardia: 677.000; ABC: 498.000) [28]. Despite efforts to correctly identify the articles that included the identified search words, we may have not located all relevant newspaper print media articles addressing PC. Finally, we acknowledge a diverse range of social and cultural factors outside of media also affect social understandings of PC [29, 30].

### Implications and future directions

The role of communication in the field of health has been widely studied in general and in relation to specific problems and policies [31–37]. Existing studies of mass media and PC indicate that the media is effective for disseminating information to overcome the lack of awareness about, and bias against, PC [38,39]. A study done in Northern Ireland identified newspapers as people’s main source of information on PC and indicated that the media is the main channel for its dissemination and promotion about health care [40].

In general, readers do not remember all the details of journalistic information, but use parts of it to build, modify or contest their own, and others’, beliefs. The media’s influence is therefore both structural and indirect [41], and future research may benefit from exploring the impact of the discourses identified here on the Spanish population’s understanding of PC. Increased understanding of the contexts in which public references to PC are currently made, the main actors who speak about it, and the dominant discourses used, may lead to the adaptation of, and improvement in communication about PC. We may then be better able to perceive possibilities of social change and transforming the meaning to individuals’ and social perceptions of PC, helping to design and implement strategies for developing these services [27,38]. Finally, increasing healthcare professionals’, patients’ and family members’ involvement in mass mediated messages about PC may minimize the power of existing ideological discourses and help strengthen a collective consciousness amongst diverse stakeholders that minimizes sensationalistic approaches and inaccurate representations [42]. Future studies could explore longitudinal changes and differences between countries in awareness, attitudes and social representations on palliative care.

## Conclusions

PC has a substantial presence in the Spanish press, generally provided by politicians in contexts of the immediate present that had little educational or experiential information. When referenced, PC was primarily connected to political and moral discourses and connected to discussion far removed from the reality of healthcare. News items on legislation of the end-of-life, where euthanasia and assisted dying were the focus of the discussion, provided a polarized discourse about PC. Instead of promoting social recognition for PC, news items on the topic may therefore be a barrier to its further development. Ensuring a stronger diversity in stakeholder representation through initiatives to promote healthcare professionals’, patients’, and family members’ messages in mainstream newspapers may be an important pathway to support comprehensive and integrated PC.

## Supporting information

S1 File List of the articles included in the analysis. (DOCX)
