# Supplementary material for: Social representation of palliative care in the Spanish printed media: A qualitative analysis
Source: PLoS One. 2019 Jan 25;14(1):e0211106. doi: 10.1371/journal.pone.0211106 (PMC6347435; doi:10.1371/journal.pone.0211106)
Supplement: S1 File — (DOCX) [file pone.0211106.s001.docx]

**S1 File. PCMedia-PlosOne-S1-V2.docx. List of the articles included in the analysis.**

**PRINTED NEWSPAPER**

***ABC***

- "En España aún mueren pacientes con unos sufrimientos evitables". (ABC; 17/01/2014)
- El Hospital Carlos III acogerá a los enfermos crónicos de media estancia. (ABC; 26/11/2013)
- Sí a la vida. (ABC; 04/04/2013)
- San Juan de Dios, una orden dedicada a los madrileños. (ABC; 16/03/2013)
- Los médicos envían a Chávez a casa por el avance de un nuevo tumor. (ABC; 01/03/2013)
- Hugo Chávez regresa a Caracas en la última fase de su enfermedad. (ABC; 19/02/2013)
- Muérase usted, por favor. (ABC; 28/01/2013)
- Hollande abre la puerta a la eutanasia con una futura ley de suicidio asistido. (ABC; 19/12/2012)
- Arranca la huelga de dos días contra la reforma sanitaria. (ABC; 26/11/2012)
- Ramón de Icaza y Zabálburu (1936-2012) Histórico consejero del Bilbao. (ABC; 25/10/2012)
- Botella inaugura un centro de enfermedades avanzadas. (ABC; 12/10/2012)
- Solidaridad familiar ante las graves dolencias. (ABC; 12/09/2012)
- La libertad condicional de enfermos incurables. (ABC; 26/08/2012)
- Conversaciones interceptadas a presos etarras revelan la farsa de su ayuno. (ABC; 16/08/2012)
- La huelga de hambre desvela tensión entre los presos de ETA. (ABC; 13/08/2012)
- Belén Ordóñez El discreto adiós de la hermana de Carmina. (ABC; 04/08/2012)
- Un "carcelero" de Ortega Lara, al hospital del San Sebastián. (ABC; 02/08/2012)
- Ver fútbol reduce el dolor de los pacientes terminales. (ABC; 13/06/2012)
- The Salvation Army visita la Fundación "La Caixa". (ABC; 06/06/2012)
- Las propuestas de los candidatos. (ABC; 06/05/2012)
- En Francia, una primera vuelta muy complicada. (ABC; 24/04/2012)
- Las cinco cosas de las que nos arrepentimos antes de morir. (ABC; 21/12/2011)
- Eutanasia y PSOE. (ABC; 30/08/2011)
- Retiran la alimentación a una enferma sin testamento vital. (ABC; 25/08/2011)
- ¿Qué hará el PP si accede al gobierno? (ABC; 08/08/2011)
- Estado de depresión. (ABC; 28/06/2011)
- "Algunos aspectos de la ley de 'muerte digna' son proeutanásicos". (ABC; 24/06/2011)
- Vida y muerte. (ABC; 13/06/2011)
- Eutanasia encubierta. (ABC; 12/06/2011)
- El gobierno da luz verde a la ley de muerte digna. (ABC; 11/06/2011)
- Jack Kervorkian El final del "doctor muerte". (ABC; 04/06/2011)
- Un proyecto de ingeniería social . (ABC; 26/05/2011)
- Eutanasia. (ABC; 22/05/2011)
- Ya hay muerte digna. (ABC; 16/05/2011)
- ¿Seguridad jurídica o paso a la eutanasia? (ABC; 14/05/2011)
- Muerte digna o eutanasia. (ABC; 14/05/2011)
- La ley de Muerte Digna no permitirá la objeción de conciencia médica. (ABC; 13/05/2011)
- Calidad de vida hasta el final. (ABC; 08/05/2011)
- El congreso languidece ante la escasa iniciativa del Gobierno. (ABC; 24/04/2011)
- Pajín anuncia para mayo la polémica ley de muerte digna. (ABC; 06/04/2011)
- Un año de agonía legislativa. (ABC; 04/04/2011)
- Un clamor por la vida. (ABC; 27/03/2011)
- Por qué acudir al acto "¡Sí a la vida!". (ABC; 25/03/2011)
- Las comunidades socialistas avivan el debate de la muerte digna y la eutanasia. (ABC; 25/03/2011)
- El miedo al dolor, el mayor temor. (ABC; 25/03/2011)
- "Nadie al borde de la muerte me ha pedido la eutanasia". (ABC; 24/02/2011)
- Francia entierra la Ley de Eutanasia / Un debate no cerrado en España. (ABC; 26/01/2011)
- Los médicos piden ayudas exprés para enfermos terminales. (ABC; 19/01/2011)
- "El debate de la eutanasia no se ha agotado con la ley de muerte digna". (ABC; 28/11/2010)
- El PSOE admite que el "caso montes" inspira su ley de la "muerte digna". (ABC; 23/11/2010)
- El gobierno se lanza a regular por ley la "muerte digna". (ABC; 20/11/2010)
- "Me sobrecoge la fortaleza de los que se enfrentan al fin de la vida". (ABC; 20/10/2010)
- Enfermos terminales atendidos 24 horas. (ABC; 17/07/2010)
- "La ELA es una enfermedad incurable y devastadora". (ABC; 31/05/2010)
- La "muerte digna", en vigor en Andalucía a finales de mes. (ABC; 08/05/2010)
- "Ahora sé lo duro que es estar en la Unidad de Dolor de un hospital". (ABC; 19/03/2010)
- El congreso rechaza abrir el debate sobre el "derecho a la eutanasia". (ABC; 10/03/2010)
- Personal del Clínico teme un "caos" en el traslado por obras. (ABC; 12/10/2009)
- Los médicos reclaman más cuidados paliativos para evitar la eutanasia. (ABC; 07/10/2009)
- "La sociedad debe unirse en un frente común contra el cáncer” (ABC; 29/09/2009)
- Máster de Bioética y Bioderecho de la Universidad Rey Juan Carlos. (ABC; 04/09/2009)
- El Parlamento catalán acuerda debatir la eutanasia. (ABC; 19/06/2009)
- Andalucía forzará a los médicos a aplicar su ley de "muerte digna". (ABC; 10/06/2009)
- Inmaculada Echavarría cumplió su deseo de ser desconectada. (ABC;10/06/2009)
- El triplete del no Passssa Nada. (ABC; 31/05/2009)
- "Por la puerta del testamento vital puede colarse la eutanasia". (ABC; 14/03/2009)
- De los cuidados paliativos a la legalización de la eutanasia. (ABC; 09/03/2009)
- "En España aún mueren pacientes con unos sufrimientos evitables". (ABC-Sevilla; 21/01/2014)
- I Curso de formación en Cuidados Paliativos. (ABC-Sevilla; 05/11/2013)
- Tres mil pacientes reciben paliativos cada año. (ABC-Sevilla; 02/11/2013)
- Redes asistenciales. (ABC-Sevilla; 12/10/2013)
- Obstinación terapéutica, un tormento al paciente terminal. (ABC-Sevilla; 22/03/2013)
- El Año Jubilar Macareno tendrá obra social y un congreso mariológico internacional. (ABC-Sevilla; 10/03/2013)
- Investigación y leyes contra el tabaco, armas letales para el cáncer de pulmón. (ABC-Sevilla; 22/11/2012)
- Arenas pide una auditoría de las facturas de la Junta. (ABC-Sevilla; 20/01/2012)
- El documental "Alma", premio del Cicus. (ABC-Sevilla; 12/11/2011)
- Los últimos días de Ramona. (ABC-Sevilla; 12/09/2011)
- Vivir con dignidad y morir en paz. (ABC-Sevilla; 06/09/2011)
- Derecho a Vivir acusa a la Junta de "dejar de alimentar" a una anciana. (ABC-Sevilla; 26/08/2011)
- "La Consejería no es nadie para dar una orden de este tipo". (ABC-Sevilla; 25/08/2011)
- Rubalcaba avisa de que su primera ley será la que regule la "muerte digna". (ABC-Sevilla; 23/08/2011)
- "Al Gobierno andaluz le hace falta la Divina Providencia para pagar sus deudas". (ABC-Sevilla; 07/08/2011)
- Cuidados paliativos, ya. (ABC-Sevilla; 22/06/2011)
- El gobierno da luz verde a la ley de muerte digna. (ABC-Sevilla; 11/06/2011)
- Un proyecto de ingeniería social . (ABC-Sevilla; 26/05/2011)
- Y se prepara el cierre de camas en el Duque del Infantado. (ABC-Sevilla; 12/05/2011)
- Calidad de vida hasta el final. (ABC-Sevilla; 08/05/2011)
- Banderas para todo. (ABC-Sevilla; 02/04/2011)
- Sevilla también se echó a la calle para protestar contra el aborto y cualquier atentado a la vida. (ABC-Sevilla; 28/03/2011)
- Por qué acudir al acto "¡Sí a la vida!". (ABC-Sevilla; 25/03/2011)
- El miedo al dolor, el mayor temor. (ABC-Sevilla; 25/03/2011)
- Pabellón vasco. (ABC-Sevilla; 03/03/2011)
- "Los adolescentes mejoran cuando se tratan en unidades pediátricas". (ABC-Sevilla; 16/02/2011)
- Manipulación semántica y hartazgo ciudadano. (ABC-Sevilla; 01/12/2010)
- "El debate de la eutanasia no se ha agotado con la ley de muerte digna". (ABC-Sevilla; 28/11/2010)
- La Parca, de nuevo en Sevilla. (ABC-Sevilla; 05/11/2010)
- La US acoge la Asamblea Estatal de Estudiantes de Medicina. (ABC-Sevilla; 25/10/2010)
- Expertas piden recursos para luchar contra el cáncer infantil. (ABC-Sevilla; 22/10/2010)
- Atención domiciliaria. (ABC-Sevilla; 12/10/2010)
- Expertos reclaman unidades de Cuidados Paliativos Pediátricos. (ABC-Sevilla; 09/10/2010)
- La "muerte digna", en vigor en Andalucía a finales de mes. (ABC-Sevilla; 08/05/2010)
- Una gran labor asistencial. (ABC-Sevilla; 26/04/2010)
- Protesta laboral por la reconversión del hospital Duque del Infantado. (ABC-Sevilla; 21/04/2010)
- Hermano Francisco Simón - El limosnero de Sevilla. (ABC-Sevilla; 17/04/2010)
- ¡Muerte digna, sí, Eutanasia, no! (ABC-Sevilla; 24/03/2010)
- El Hospital San Lázaro, lleno de gritas y con riesgo de desprendimientos. (ABC-Sevilla; 22/03/2010)
- Aprobada la ley de muerte digna pese a faltar habitaciones individuales para casos terminales. (ABC-Sevilla; 18/03/2010)
- El 20% del área hospitalaria Macarena cuenta con habitaciones individuales. (ABC-Sevilla; 02/02/2010)
- Los médicos reprochan a la Junta que vete la objeción de conciencia a la muerte digna. (ABC-Sevilla; 18/11/2009)
- Joaquín Torres releva a Pilar Serrano en la gerencia del Virgen Macarena. (ABC-Sevilla; 05/11/2009)
- Día mundial de los cuidados paliativos. (ABC-Sevilla; 12/10/2009)
- Aval del Gobierno a la Ley de muerte digna. (ABC-Sevilla; 08/10/2009)
- "Es una barbaridad querer configurar al hombre a base de mayorías". (ABC-Sevilla; 22/06/2009)
- El Parlamento catalán acuerda debatir la eutanasia. (ABC-Sevilla; 19/06/2009)
- Los médicos reclaman poder opinar sobre la Ley de Muerte Digna. (ABC; 17/06/2009)
- La ley de "muerte digna" excluye el derecho a objeción de conciencia médica. (ABC-Sevilla; 10/06/2009)
- El triplete del no Passssa Nada. (ABC-Sevilla; 31/05/2009)
- Sevilla concederá la Medalla de la Ciudad a los cuatro alcaldes de la democracia. (ABC-Sevilla; 11/05/2009)
- "Las mujeres hacemos falta también en la Iglesia; con el tiempo habrá obispas. (ABC-Sevilla; 31/03/2009)
- Premio a la mejor comunicación. (ABC-Sevilla; 25/03/2009)
- El hospital de San Lázaro se adapta a la "inminente" Ley de Muerte Digna. (ABC-Sevilla; 10/03/2009)
- Nueva unidad de cuidados paliativos en San Lázaro. (ABC-Sevilla; 10/03/2009)
- La voz de Sevilla. (ABC-Sevilla; 07/03/2009)
- Recibe un certificado de calidad. (ABC-Sevilla; 11/02/2009)
- La inversión privada en sanidad supera ya la de las obras paralizadas de la Junta. (ABC-Córdoba; 13/11/2013)
- Satse denuncia la eliminación de puestos de trabajo. (ABC-Córdoba; 27/06/2013)
- La obra de la residencia del Sociosanitario empezará en mayo. (ABC-Córdoba; 31/01/2012)
- Cuidados paliativos para 1.400 personas. (ABC-Córdoba; 26/01/2012)
- Satse denuncia la supresión de los cuidados paliativos en la comarca. (ABC-Córdoba; 22/11/2011)
- San Juan de Dios inicia la obra que modernizará sus servicios. (ABC-Córdoba; 26/10/2011)
- San Juan de Dios ofrecerá tratamiento oncológico completo. (ABC-Córdoba; 08/07/2011)
- Un millar de personas se congrega en defensa de la vida. (ABC-Córdoba; 27/03/2011)
- Una plataforma demanda políticas para embarazadas y enfermos. (ABC-Córdoba; 25/03/2011)
- San Juan de Dios se amplía en 80 camas, ocho quirófanos y el triple de consultas. (ABC-Córdoba; 09/03/2010)
- Salud insiste en no regular la objeción de conciencia de los médicos en la Ley de Muerte Digna. (ABC-Córdoba; 09/07/2009)

***El País***

- Un paso más hacia una muerte digna. (El País; 01/06/2013)
- El primer centro privado holandés para eutanasia tiene lista de espera. (El País; 17/03/2013)
- Francia abre la puerta al suicidio asistido de enfermos terminales. (El País; 19/12/2012)
- "Si me quedase embarazada de otro bebé tan enfermo iría a abortar fuera". (El País; 26/07/2012)
- Peter Goodwin, médico que abrió el camino al final digno. (El País; 23/03/2012)
- "No agradezco al médico estar vivo". (El País; 30/01/2012)
- "Lo que Pedro pedía era una eutanasia y eso no es posible". Regular el bien morir, tarea pendiente. (El País; 23/12/2011)
- "Vivo en una cárcel que se estrecha". Un agujero en la ley. (El País; 22/12/2011)
- Una guía médica regula la sedación paliativa. (El País; 11/11/2011)
- Lo social y las distracciones. (El País; 29/10/2011)
- Las cesiones del gobierno. (El País; 14/08/2011)
- Enfermos terminales y cuidados paliativos. (El País; 09/08/2011)
- Madrid elude su deber de garantizar paliativos a la anciana de Leganés. (El País; 03/08/2011)
- No hay muerte digna para Carmen López. (El País; 02/08/2011)
- Denunciada una residencia por no aplicar paliativo a una anciana. (El País; 02/08/2011)
- Cuidado o tratamiento. (El País; 02/08/2011)
- Los médicos de cuidados paliativos apoyan la ley. (El País; 17/05/2011)
- Morir sin dolor. (El País; 16/05/2011)
- ¿Quién teme a la eutanasia? (El País; 15/05/2011)
- Cala la cultura de la humanización del fallecimiento. (El País; 14/05/2011)
- El consejo de ministros aprueba hoy la ley de muerte digna. (El País; 13/05/2011)
- Insultos para la audiencia. (El País; 27/04/2011)
- El paciente sufre las esperas y la descoordinación. (El País; 15/04/2011)
- Más geriatras para descongestionar las urgencias de los hospitales. (El País; 29/03/2011)
- Fumar hasta el final. (El País; 21/03/2011)
- El PSOE intenta cerrar la puerta a la eutanasia en el congreso. (El País; 09/02/2011)
- Francia reabre el debate de la eutanasia más allá de las ideologías. España limita la discusión a los cuidados paliativos. (El País; 26/01/2011)
- Un cáncer del páncreas curado gracias al trasplante a un ratón. (El País; 11/01/2011)
- El PP bautiza 2010 como el "año horrible" de la sanidad. (El País; 21/12/2010)
- La lista de espera quirúrgica baja a 61 días, la menor registrada. (El País; 10/12/2010)
- Jáuregui plantea a Rouco que la Iglesia respete la soberanía popular. (El País; 08/12/2010)
- Quiero morir en casa. ¿Puedo? (El País; 03/12/2010)
- Morir con dignidad. (El País; 20/11/2010)
- Zapatero retoma el impulso social para agotar la legislatura en 2012. (El País; 20/11/2010)
- Una ley para la muerte digna blindará la acción de los médicos. (El País; 20/11/2010)
- Un 29% de los dirigentes médicos se declara a favor de la eutanasia. (El País; 18/11/2010)
- "Haré cumplir la ley del tabaco con los instrumentos del Estado" . (El País; 12/11/2010)
- "¿Verdades? Solo cuando las pida el enfermo". (El País; 21/09/2010)
- España suspende en paliativos. (El País; 16/08/2010)
- Nada más allá de los cuidados paliativos. (El País; 19/06/2010)
- Sanidad centralizará las compras de vacunas para la gripe. (El País; 01/06/2010)
- La subjetividad define qué enfermo es terminal. (El País; 06/04/2010)
- Si hay que morir, mejor saberlo. (El País; 22/03/2010)
- Paliativos contra el dolor sordo. (El País; 02/03/2010)
- Poca atención a los niños en cuidados paliativos. (El País; 10/10/2009)
- Unos 2000 enfermos terminales al año piden la eutanasia. (El País; 07/10/2009)
- La justicia de Australia acepta que se deje de alimentar a un tetrapléjico. (El País; 15/08/2009)
- 56.000 personas registran su voluntad de morir dignamente. (El País; 13/08/2009)
- La eutanasia filmada que nunca existió. (El País; 21/06/2009)
- Aún se muere sufriendo . (El País; 21/06/2009)
- Lamela: "Jamás insinué que hubiera eutanasia en Leganés". (El País; 18/06/2009)
- El derecho a morir en paz. (El País; 17/06/2009)
- Asturias y Cataluña se interesan por la regulación andaluza de la muerte digna. (El País; 16/06/2009)
- El PP sólo apoyará la ley de muerte digna si respeta las "creencias”. Explíquenselo a la ministra. (El País; 11/06/2009)
- Andalucía regula por ley el derecho del enfermo terminal a la sedación. (El País; 10/06/2009)
- Guía audiovisual para atención a enfermos terminales. (El País; 09/06/2009)
- Sólo hay 400 unidades de paliativos para 200.000 enfermos terminales. (El País; 29/05/2009)
- "Los males de la sanidad no son por falta de médicos". (El País; 12/05/2009)
- Muerte digna. (El País; 23/04/2009)
- "El aborto está socialmente superado". (El País; 20/04/2009)
- Veinte meses con muchas propuestas y poco que hacer. (El País; 08/04/2009)
- ¿Dónde está el Dr. House? (El País; 13/03/2009)
- España también deja rendijas para una Eluana. (El País; 13/02/2009)
- Víctima. (El País; 02/02/2009)
- El corto camino al Oscar . (El País; 09/01/2009)

***El Mundo***

- España, huérfana de cuidados paliativos. (El Mundo; 17/02/20014)
- Bélgica legaliza la eutanasia infantil. (El Mundo; 14/02/2014)
- La metástasis del sistema alcanza a los "con papeles". (El Mundo; 17/06/2013)
- La Unidad de Abrazos Paliativos. (El Mundo; 02/04/2013)
- El PSOE agita viejos fantasmas. (El Mundo; 07/02/2012)
- Investigan una millonaria web cedida a un ex alto cargo de Vara. (El Mundo; 06/11/2011)
- Joe Frazier tiene cáncer de hígado. (El Mundo; 06/11/2011)
- La quiniela de… Federico Trillo. (El Mundo; 04/11/2011)
- El rastro del fantasma de Lady Di. (El Mundo; 27/04/2011)
- El sentido de la muerte y de la vida. (El Mundo; 02/02/2011)
- Las unidosis llegarán a España en enero. (El Mundo; 10/12/2010)
- Rubalcaba dice que dentro de cuatro meses se aprobará la Ley sobre la Muerte Digna. (El Mundo; 20/11/2010)
- 37 millones para cuidados paliativos. (El Mundo; 17/07/2010)
- Rivas promueve su Asesoría por una Muerte Digna. (El Mundo; 01/11/2009)
- Reforma legal sobre el suicidio asistido. (El Mundo; 30/10/2009)
- El sacerdote al que pretendió la CIA. (El Mundo; 27/10/2009)
- Dulce, odioso, desafiante, genial. (El Mundo; 30/08/2009)
- La enfermera de Rayan. (El Mundo; 18/07/2009)
- La muerte digna. (El Mundo; 19/06/2009)
- Andalucía aprueba la primera ley española sobre muerte digna. (El Mundo; 10/06/2009)
- Ignacio graba el adiós. (El Mundo; 29/03/2009)
- Morir muerto. (El Mundo; 01/03/2009)
- Especializados en mitigar el dolor de los que más sufren. (El Mundo; 18/01/2009)

***La Vanguardia***

- La Universitat de Vic prevé abrir facultad de Medicina. (La Vanguardia; 02/12/2013)
- ¿Es necesario sufrir para morir? (La Vanguardia; 27/10/2013)
- Stephen Hawking apoya el derecho al suicidio asistido. (La Vanguardia; 18/09/2013)
- Salut quiere dar cuidados paliativos a los enfermos crónicos graves. (La Vanguardia; 23/01/2013)
- Baleares rectifica y decide mantener dos hospitales. (La Vanguardia; 25/11/2012)
- "El dolor no tiene sentido, lo único que sí lo tiene es intentar mitigarlo". (La Vanguardia; 28/06/2012)
- Down+Alzheimer. (La Vanguardia; 06/02/2012)
- "Los pacientes oncológicos atendidos en equipos multidisciplinares tienen mayor supervivencia". (La Vanguardia; 04/02/2012)
- Orfandad 'queer' -Paula Ettelbrick (1955-2011). (La Vanguardia; 05/11/2011)
- Zapatero y Rajoy se miden en un momento dramático para Europa. (La Vanguardia; 28/06/2011)
- Los obispos instan a desobedecer la ley de muerte digna si no se modifica. (La Vanguardia; 28/06/2011)
- Curarse un cáncer a los 80. (La Vanguardia; 14/06/2011)
- El cardenal Rouco afirma que la ley de la muerte digna "no es eutanasia" . (La Vanguardia; 27/05/2011)
- Los enfermos terminales deberán disponer de habitación individual. (La Vanguardia; 14/05/2011)
- El Gobierno busca votos con la ley de muerte digna antes del 22-M. (La Vanguardia; 30/04/2011)
- La vida en campaña. (La Vanguardia; 08/04/2011)
- Los obispos se centran ahora en arremeter contra la eutanasia. (La Vanguardia; 17/03/2011)
- La ley de muerte digna seguirá los pasos de la norma andaluza. (La Vanguardia; 27/12/2010)
- Los cuidados paliativos se extienden también a los niños . (La Vanguardia; 10/12/2010)
- El cardenal Amigo defiende los cuidados paliativos. (La Vanguardia; 23/11/2010)
- La muerte digna, por ley. ¿Qué pesa más, el temor a sufrir o a morir? (La Vanguardia; 20/11/2010)
- ¿Qué pesa más el temor a sufrir o a morir? (La Vanguardia; 20/11/2010)
- La muerte digna, por ley. (La Vanguardia; 20/11/2010)
- El gobierno lanza una ofensiva de leyes hasta junio. (La Vanguardia; 20/11/2010)
- Clínica Coroleu. (La Vanguardia; 11/11/2010)
- Doctor, ¿me muero? (La Vanguardia; 03/10/2010)
- La sociedad por delante. (La Vanguardia; 28/08/2010)
- El cáncer colorrectal se consolida como el más frecuente en Catalunya. (La Vanguardia; 13/07/2010)
- "¡Intentaré que la muerte me encuentre bien vivo!". (La Vanguardia; 10/04/2010)
- Aprende a vivir, lo que dure. (La Vanguardia; 30/11/2009)
- "Hay que entender qué quiere el paciente". (La Vanguardia; 30/11/2009)
- Apoyo integral a personas con enfermedades avanzadas. 08/11/2009)
- Todos contra el Alzheimer. (La Vanguardia; 26/08/2009)
- Muere la anciana que inició una huelga de hambre para que le aplicasen la eutanasia. (La Vanguardia; 02/04/2009)
- Ofensiva para ampliar el apoyo a pacientes terminales. (La Vanguardia; 28/03/2009)
- Sebastià Serrano invita en su nuevo libro a descubrir el papel de los sentidos. (La Vanguardia; 26/03/2009)
- Decisiones personales. (La Vanguardia; 15/02/2009)
- No sólo de curas vive el enfermo terminal. (La Vanguardia; 12/01/2009)
